# Unlocking Anion Reduction of Lithium Perchlorate via Electrochemically Coupled Oxygen Atom Transfer

**DOI:** 10.1021/jacs.5c18724

**Published:** 2026-07-14

**Authors:** Julian F. Baumgärtner, Archita Vijay, Matthias Klimpel, Dmitry Chernyshov, Wouter van Beek, Maksym V. Kovalenko, Kostiantyn V. Kravchyk

**Affiliations:** † Laboratory of Inorganic Chemistry, Department of Chemistry and Applied Biosciences, 27219 ETH Zürich, Zürich CH-8093, Switzerland; ‡ Laboratory for Thin Films and Photovoltaics, Empa—Swiss Federal Laboratories for Materials Science & Technology, Dübendorf CH-8600, Switzerland; § Swiss−Norwegian Beam Lines at the European Synchrotron Radiation Facility, Grenoble 38000, France

## Abstract

The growing demand for high energy density electrochemical energy storage necessitates energy vectors that maximize the number of electrons transferred per formula unit of active material. Herein, we introduce electrochemically coupled oxygen atom transfer (OAT) as a new paradigm to harness the energy of p-block oxoanions in a Li–metal solid-state battery. Using carbon-supported Fe nanoparticles in a dual role of OAT catalyst and conversion-type cathode active material, we demonstrate the eight-electron anion reduction of ClO_4_^–^ at >50% conversion, delivering a capacity of 1150 mA h g^–1^ and an energy density of 1950 W h kg^–1^. We further demonstrate strategies to enhance the energy density at the electrode level, establishing a foundation for oxoanion-based anion redox in battery systems.

## Graphical abstract

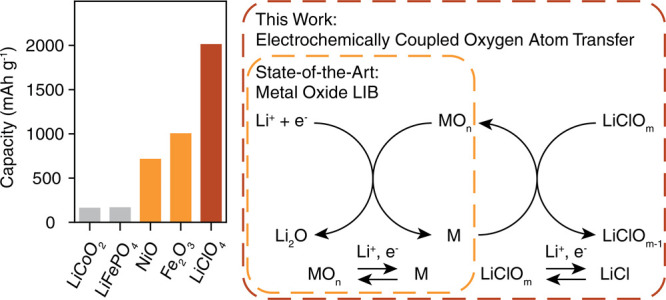

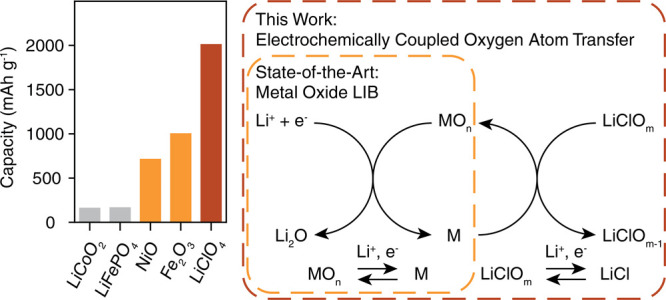

## Introduction

As the demand for electrochemical storage systems continues to grow, identifying alternative energy vectors that enable batteries with increased energy density has become essential. Currently employed Lithium-ion batteries (LIBs) rely on electrodes, in which a host structure, e.g. a transition metal oxide or graphite enables rapid (de)­intercalation of Li-ions with concomitant electron transfer. Yet to ensure structural stability of the active material upon cycling, only ca. 1 Li per transition metal (TM) or C_6_ unit is transferred, accompanied by a single electron redox on the TM or C_6_ unit, which severely limits the charge-storage capacity of such intercalation-type active materials.

To overcome these limitations, metallic anodes and conversion-type cathode active materials (CAMs) are being explored as next-generation electrode materials. Conversion-type CAMs involve multielectron TM redox or nonmetal redox (e.g., sulfur, oxygen), which enables the transfer of multiple electrons per redox center by foregoing the need for a crystalline host (Figure 1a).^1^ All of these chemistries have in common, that their aim is to maximize the theoretically achievable energy density by maximizing the number of electrons transferred while minimizing the molecular weight of the respective material. A class of CAMs that is almost entirely neglected in this context are the p-block peroxoanions, such as N^+V^O_3_^–^/N^3–^, P^+V^O_4_^3–^/P^3–^, S^+VI^O_4_^2–^/S^2–^, and Cl^+VII^O_4_^–^/Cl^–^, even though they can formally undergo eight-electron reduction at very low molecular weights. For instance, LiClO_4_ offers a theoretical capacity of 2015 mA h g^–1^_LiClO4_. Owing to the high electronegativity of Cl, the Cl^+VII^O_4_^–^/Cl^–^ couple is expected to deliver a high lithiation potential of ∼4.4 V based on the Latimer diagram,^2^ resulting in a gravimetric energy density of nearly ∼5800 Wh kg^–1^ against a Li metal anode, vastly surpassing all current LIB chemistries, including Li–S and Li–air batteries (Figure 1a).^3^

**Figure 1:**
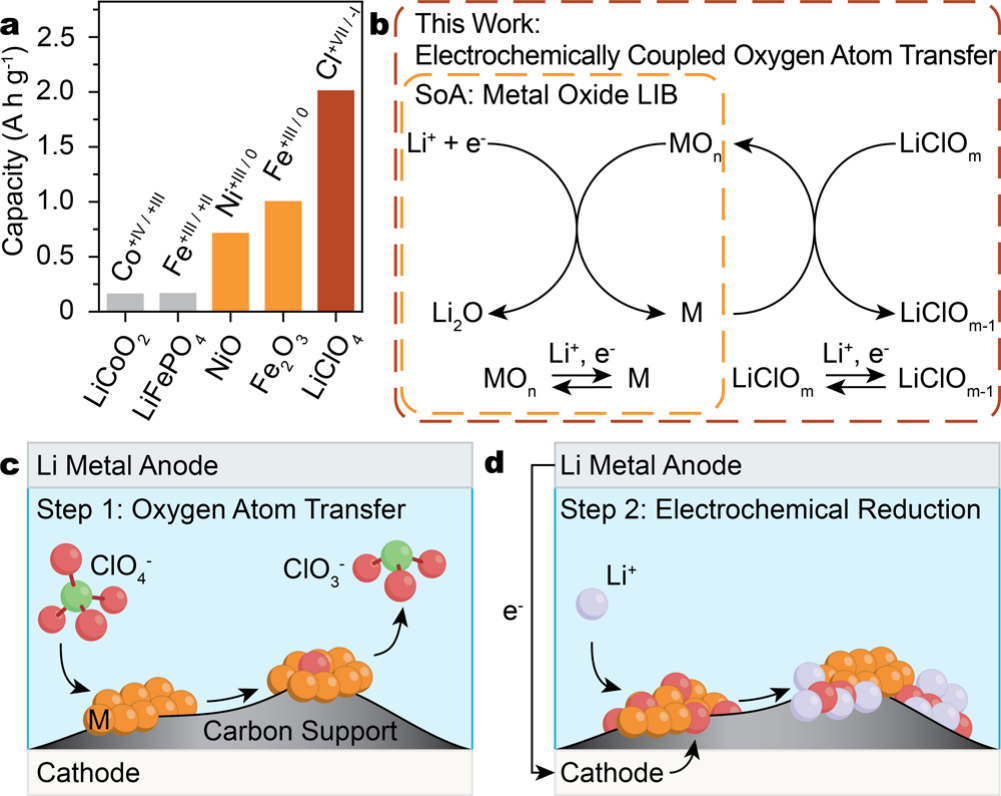
Electrochemically coupled oxygen atom transfer (OAT) of LiClO_4_. (a) Theoretical capacity of state-of-the-art intercalation CAMs (gray), conversion-type TMO_*x*_ CAMs (orange), and LiClO_4_ (brown) (b) operating principle of conventional conversion-type Li-ion batteries based on spatially separated redox reactions between Lithium metal anodes and metal oxide cathodes, and the electrochemically coupled OAT based on LiClO_4_ proposed in this work. (c, d) Working principle of the proposed electrode. In a first step, ClO^*n*–^ is chemically reduced by a transition metal catalyst on the electrode via OAT (c), which then subsequently undergoes electrochemical lithiation via a conversion-type mechanism (d).

Despite their massive theoretical energy gains, p-block peroxoanions have rarely been regarded as viable CAMs, presumably due to the difficulty of direct electrochemical reduction of anions on negatively polarized electrodes. Even if their strong Coulombic repulsion would be overcome, e.g. by employing molten oxoanion salt electrolysis, the formation of highly reactive radical anions would further require impractically high overpotentials. To the best of our knowledge, only a single report has considered oxoanion redox in a battery configuration, but was limited to two-electron NO_3_^–^/NO_2_^–^ reduction, offering a modest theoretical capacity of 647 mA h g^–1^.^4^

We reasoned that these challenges may be overcome using a bioinspired oxygen atom transfer (OAT) electrode. For instance, oxoanion redox is routinely achieved in biological systems via OAT mediated by TMs in the oxidoreductase enzymes, bypassing direct electron transfer.^5,6^ In an OAT, Cl is formally reduced, while the catalyst taking up the O is oxidized.^7^ For this reason, heterogeneous electrocatalysis routinely employs OAT catalysts such as TM nanoparticles (NPs), for instance in nitrate reduction,^8^ or in wastewater treatment of oxoanions.^2,5,9^ Notably, many of these same metals, e.g. Fe and Ni, also function as conversion-type CAMs in LIBs.^10^

Building on these insights, we reasoned that TM NPs could serve a dual function of catalyzing oxoanion OAT, and subsequently act as an electrode which can be reduced by electrons with concomitant Li^+^-ion uptake (Figure 1b–d). In this concept, ClO_4_^–^ would be delivered via a liquid catholyte, while the solid cathode provides both catalytic and electrochemical functionality, resembling a hybrid between a solid-state and redox-flow battery.

Herein we report the electrochemical reduction of LiClO_4_ in a primary solid-state Li-ion battery (SSB), using a cathode composed of TM NPs supported on porous carbon (M@C). By screening various TMs, we identify earth-abundant Fe as a highly effective catalyst, achieving >50% conversion of LiClO_4_, and delivering a capacity of 1150 mA h g^–1^_LiClO4_ at a practical discharge voltage of 2.2 V. We further demonstrate strategies to enhance the energy density at the electrode level, establishing a foundation for oxoanion-based anion redox in battery systems.

## Results and Discussion

### Synthesis of Carbon-Supported Transition Metal Nanoparticle Catalysts

An effective electrocatalyst must facilitate both the thermochemical OAT, as well as the subsequent electrochemical reduction of the resulting metal oxide, while also ensuring rapid transport of Li^+^, ClO_4_^–^ and electrons to the active site. To support these functions, the catalyst support must be highly porous and electronically conductive. In this work, carbon was chosen as the catalyst support owing to its low density, high electronic conductivity, and electrochemical stability. Meanwhile, the TM catalyst should be embedded onto the support in nanoparticulate form since NPs have an increased fraction of surface-exposed TM atoms available for the OAT, and shorten the Li-ion and electron diffusion pathways during subsequent lithiation.

The synthesis of M@C (M = Fe, Co, Ni) catalysts was adapted from a reported route for FeF_3_@C (Methods, Figures 2a and S1),^11^ and performed as follows: Aqueous solutions of metal nitrates and poly­(vinylpyrrolidone) (PVP) were dried at 80 °C to yield composite precursors, which were carbonized at 700 °C under Ar. During the carbonization, the metal salts are reduced to metallic TM NPs with concomitant gas evolution from PVP and nitrate decomposition, which generates a highly porous carbon support.

**Figure 2:**
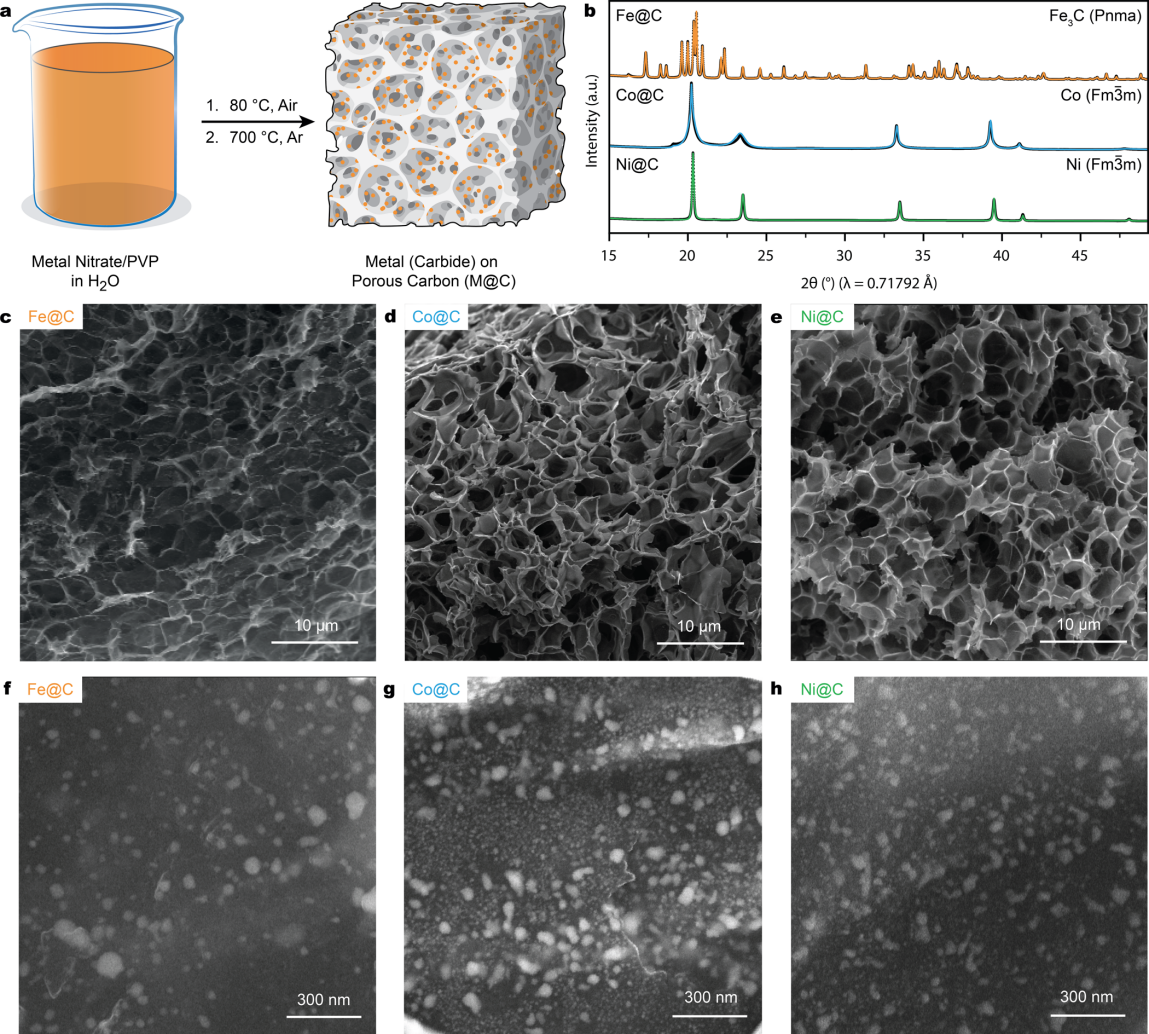
Synthesis of carbon-supported transition metal nanoparticle catalysts (TM@C). (a) Schematic representation for the preparation of TM@C catalysts involving a sol–gel synthesis of PVP and TM­(NO_3_)_*x*_ precursors, followed by high-temperature carbonization of the PVP and carbothermal reduction of the TM. (b) SXRD refinements of Fe@C, Co@C and Ni@C. (c–h) SEM micrographs of Fe@C (c, f), Co@C (d, g) and Ni@C (e, h).

The M@C catalyst was composed of large carbon monoliths (50–500 μm) with a porous, honeycomb-like architecture and nanometer-thin walls decorated with finely dispersed 10–50 nm TM NPs, as shown in the SEM micrographs (Figures 2c–h and S2). Synchrotron X-ray diffraction (SXRD) further confirmed the formation of reduced TM phases within the carbon matrix (Figures 2b and S3, Table S1).

Fe@C contained Fe_3_C (S.G. *Pnma*) alongside a minor (<7 wt %) Fe (S.G. *Im*3̅*m*) phase, consistent with the products of carbothermal reduction of iron oxide.^12^ Meanwhile, Co@C and Ni@C yielded phase-pure metals (S.G. *Fm*3̅*m*), but with substantial structural disorder, manifested as stacking faults or twinning along the (111) planes (ca. 15% in Co and 2.5% in Ni, Figure S3). Importantly, no crystalline metal oxide residues were observed in the SXRD patterns. The absence of a characteristic metal oxide atom pair correlation in the synchrotron X-ray total scattering patterns on the same samples further excluded any quantitative amorphous metal oxide residues (Figure S4). The TM content, determined by combustion analysis to the stoichiometric metal oxide, was 70–80 wt % for all samples. This synthetic approach thus provides access to structurally well-defined M@C catalysts with high metal content for a range of TMs, suitable for further probing their electrocatalytic properties toward LiClO_4_.

### Preparation of SSBs Containing TM@C Cathodes Infiltrated with LiClO_4_

To harness the Gibbs free energy of LiClO_4_ reduction electrochemically, the liquid LiClO_4_-containing catholyte must be spatially separated from the Li metal anode. Whereas conventional separators in LIBs are permeable to liquid electrolytes, the advent of solid-state electrolytes (SSE) has enabled the exploration of soluble redox-active species in hybrid SSB configurations.^13^ We therefore designed a hybrid SSB comprising a dense SSE as the separator, and a cathode containing the TM@C catalyst infiltrated with a liquid LiClO_4_-containing catholyte.

Li_7_La_3_Zr_2_O_12_ (LLZO) was selected as the SSE because of its high ionic conductivity and chemical stability against metallic Li. Dense LLZO SSE pellets were fabricated by compressing LLZO powder into green-body pellets, followed by ultrafast sintering (Methods, Figure 3b,c).^14,15^ Cathodes containing the M@C catalyst were prepared by mechanochemically mixing a slurry of M@C, carbon black (CB) and pVdF binder, followed by tape casting onto Al current collectors under inert atmosphere. To preserve the monolithic porous structure of the M@C catalyst, low-speed mixing was employed. This method enabled the fabrication of thick electrodes (ca. 100 μm) without structural degradation of the M@C catalyst (Figures 3a,d,e and S5).

**Figure 3:**
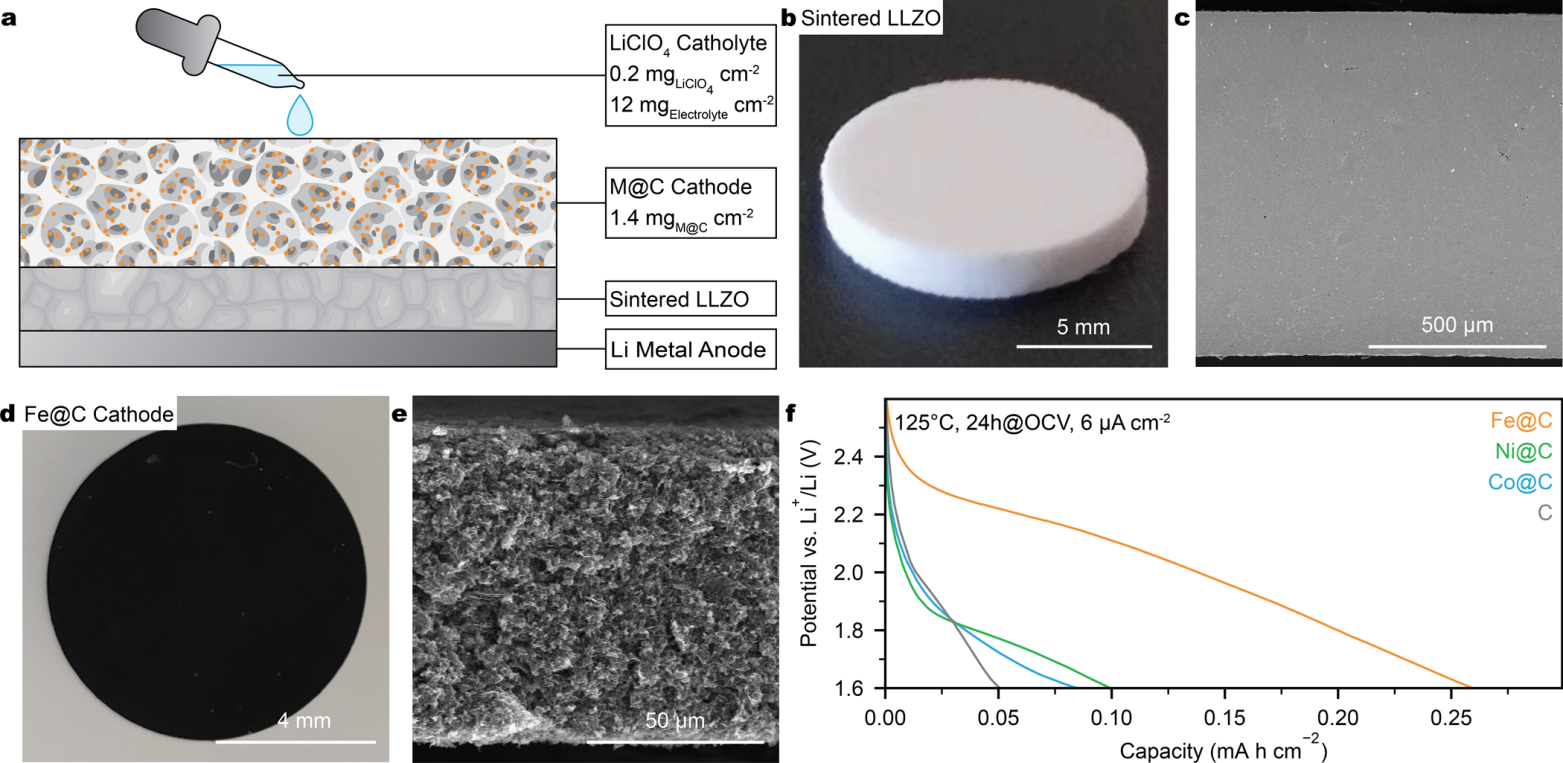
Preparation of SSBs containing TM@C cathodes infiltrated with LiClO_4_. (a) Schematic representation of the SSB architecture. (b–e) Optical image (b, d) and cross-section SEM (c, e) of a sintered LLZO pellet (b, c) and a Fe@C cathode (d, e). (f) Discharge profile of SSBs with cathodes containing different kinds of TM@C catalysts.

The M@C cathodes were subsequently infiltrated with a LiClO_4_ (0.2 mg cm^–2^_Cathode_) dissolved in 1-Butyl-1-methylpyrrolidinium bis­(fluorosulfonyl)­imide (Pyr_1,4_TFSI) (12 mg cm^–2^_Cathode_). Pyr_1,4_TFSI was chosen as the catholyte solvent because of its negligible vapor pressure at the elevated temperatures used during electrochemistry, wide electrochemical stability window, and good chemical compatibility with LLZO.^14,16^

To assess the electrochemical performance, full cells were assembled with the LLZO separator and a Li metal anode, and heated to 125 °C for 24 h to promote the thermochemical reduction of ClO_4_^–^, followed by a discharge to 1.6 V to reduce the in situ formed metal oxide NPs. The cutoff voltage was chosen to prevent excessive Pyr_1,4_TFSI electrolyte decomposition (Figure S6).

Due to the strongly oxidizing nature of perchlorate salts and the elevated operating temperature employed in this study, the present cell configuration should be regarded as a proof-of-concept platform rather than an intrinsically safe battery system. All experiments involving LiClO_4_ were therefore conducted under inert atmosphere and on small scales (<10 mg) (Methods).

Among the different M@C cathodes, Fe@C displayed the highest areal capacity (0.25 mAh cm^–2^) and the highest discharge voltage (2.2 V) at comparable catalyst and LiClO_4_ loadings. Ni@C and Co@C cathodes also displayed some capacity (0.08 and 0.1 mAh cm^–2^ respectively), but at a lower voltage (1.8 V). We attribute the higher reduction potential of the Fe@C containing cell to the accessibility of Fe^+III^ (*vide infra*), while the + III oxidation state is inaccessible for Ni and Co-containing samples. A control electrode containing LiClO_4_, and a carbon matrix without TM NPs confirmed that the presence of TMs is necessary for the ClO_4_^–^ reduction. Based on its superior activity, Fe@C was selected for further mechanistic studies.

### Mechanism of Electrochemically Coupled Oxygen Atom Transfer

In the proposed mechanism, the overall electrochemical reduction of LiClO_4_ proceeds via two sequential steps: (i) thermochemical reduction of LiClO_4_ on TM NPs to form LiClO_4‑n_ and TMO_*x*_, followed by (ii) electrochemical reduction of TMO_*x*_ by electrons accompanied by Li-ions to form TM and Li_2_O. To verify the initial thermochemical reduction of LiClO_4_, Fe@C cathodes were infiltrated with either (i) LiClO_4_ in Pyr_1,4_TFSI or (ii) LiTFSI in Pyr_1,4_TFSI as a reference cathode, and heated to 200 °C. Complete reduction of LiClO_4_ should yield solid LiCl, which is insoluble in polar solvents, unlike the intermediate LiClO_4‑*n*_ species, and can thus be detected after rinsing the electrode with dimethyl carbonate to remove residual catholyte.

The pronounced Cl signal from LiCl in the SEM/EDX maps and integrated spectra confirmed a full eight-electron reduction of LiClO_4_ in the LiClO_4_-infiltrated Fe@C samples (Figure 4a–e). In contrast, no Cl was detected in the control samples with LiTFSI, ruling out background contamination (Figure 4e). The Cl signal is distributed across the carbon matrix, with no other spatially correlated detectable metallic species. This strongly suggests Li-ions as the counterions, consistent with LiCl formation upon LiClO_4_ reduction (Figure 4d). Meanwhile, oxidation of the Fe NPs to FeO_*x*_ was observed through the spatial overlap of Fe and O signals (Figure 4b,c), confirming the OAT mechanism and, excluding noncatalytic thermal ClO_4_^–^ decomposition as the source of LiCl, since the accompanied O_2_ evolution would not result in a detectable O signal. XRD of the same Fe@C samples further corroborated the formation of spinel-type Fe_3_O_4_ upon exposure to LiClO_4_, while the Fe_3_C phase diminished in intensity (Figure 4f), providing further evidence for the proposed thermochemical OAT mechanism. We note that the formation of spinel-type Fe_3_O_4_ is not observed at lower temperatures (Figure S7).

**Figure 4:**
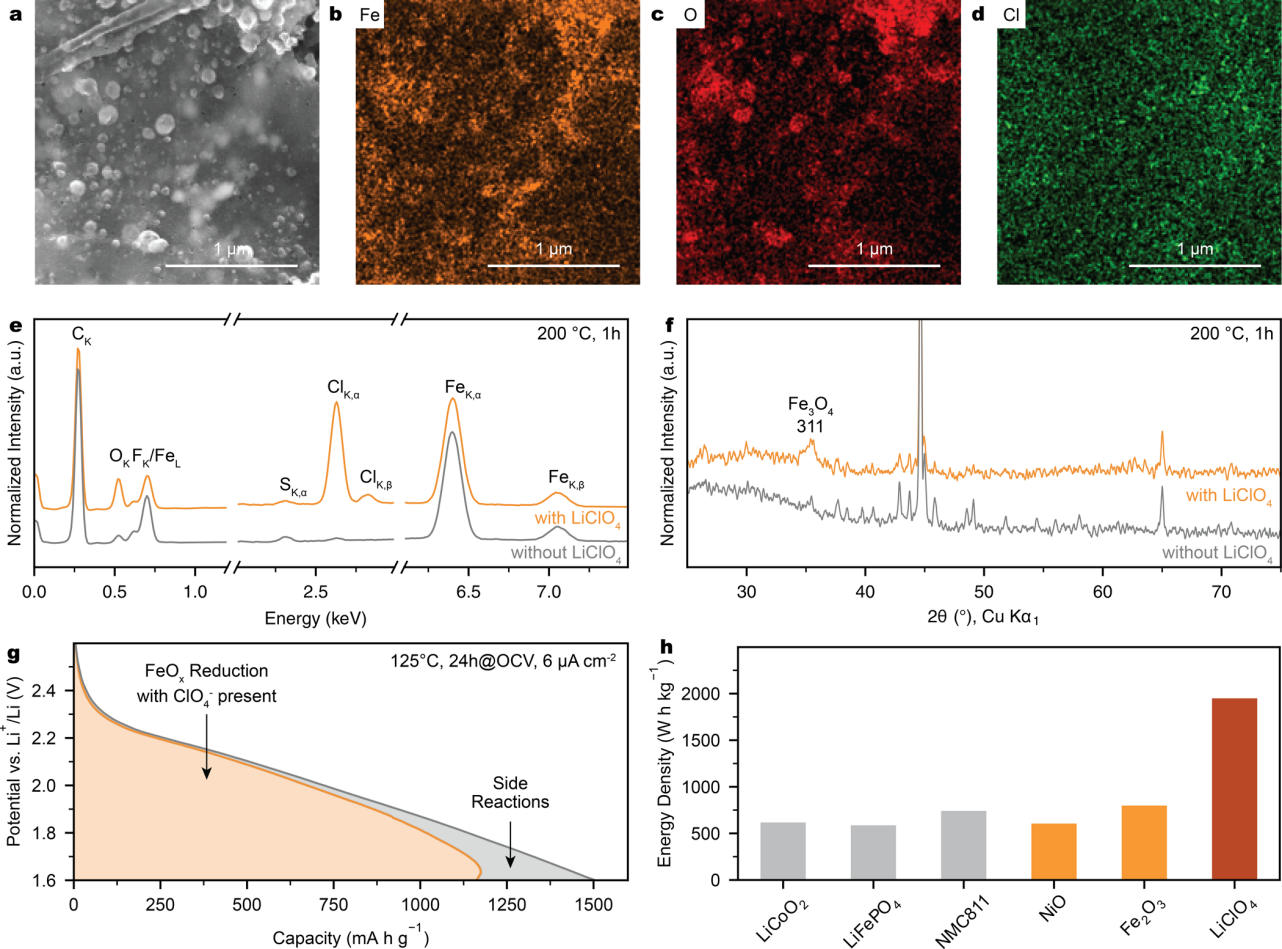
Mechanism of electrochemically coupled oxygen atom transfer. (a–f) Characterization of Fe@C cathodes infiltrated after thermochemical reduction of LiClO_4_ at 200 °C for 1 h. (a–d) SEM micrographs (a) and EDX maps (b–d) of Fe@C cathodes of Fe@C cathodes after reduction of LiClO_4_. (e, f) EDX spectra (e) and XRD patterns (f) of the same Fe@C cathodes shown in (a–d) infiltrated with Pyr_1,4_TFSI with (orange) and without (gray) LiClO_4_. (g) Discharge profile of SSBs with Fe@C cathodes containing LiClO_4_. The orange area indicates the contribution from FeO_*x*_ reduction in the presence of LiClO_4_, whereas the gray area indicates the excess capacity from side reactions. (h) Capacity of different electrochemical storage vectors.

The fact that the FeO_*x*_ NPs retained their size and morphology while LiCl is dispersed across the carbon matrix indicates that the LiClO_4_ reduction likely proceeds heterogeneously on the Fe NPs without Fe dissolution, but that the formed LiCl product is at least partially soluble in the catholyte and can therefore subsequently migrate away from the FeO_*x*_ NPs, leaving the catalytic sites accessible for further LiClO_4_ reduction.

A minor S signal was detected in both LiClO_4_- and LiTFSI-infiltrated samples, attributed to partial thermal decomposition of the TFSI^–^ anion present in the catholyte. This raised the possibility that some of the observed electrochemical capacity may be caused by S reduction from parasitic TFSI^–^ reduction. To quantify the parasitic capacity contribution, the two cathodes containing either LiClO_4_ or LiTFSI in Pyr_1,4_TFSI were assembled in full cells and discharged under the same conditions used for the initial electrochemical screening. The electrochemical capacity contribution from LiClO_4_ was isolated by subtracting the voltage profile of the LiClO_4_-free cell from that of the LiClO_4_-containing cell (Figures 4g and S8). Over 75% of the capacity originated from ClO_4_^–^ reduction, and, due to its higher discharge voltage, ClO_4_^–^ reduction accounted for ca. 90% of the total energy. This corresponds to over 55% LiClO_4_ conversion, and a capacity of 1150 mA h g^–1^_LiClO4_, over six times higher than any state-of-the-art intercalation-type Li_*x*_TMO_2_ electrode.

## Achievable Energy Densities of Perchlorate-Fueled Cathodes

The SSB prototype cell demonstrated an outstandingly high gravimetric capacity of 1150 mA h g^–1^_LiClO4_, resulting in an energy density of 1950 W h kg^–1^ against a Li metal anode (Figure 4h). Yet, the capacity and energy density with respect to the total mass of the cathode was only 10 mA h g^–1^_Cathode_ and 22 W h kg^–1^.

To understand, how the energy density could be improved on an electrode level, a sensitivity analysis was performed, where the energy density was calculated depending on (i) the ClO_4_^–^ fraction in the catholyte (*x*_ClO4‑_ in catholyte in mol %_Anions_), (ii) the Li^+^ fraction in the catholyte (*x*_Li+_ in catholyte in mol %_Cations_), (iii) the ClO_4_^–^ conversion fraction (in mol %), (iv) the porosity of the carbon support, i.e. the fraction of the liquid catholyte relative to the entire cathode (*x*_Catholyte_ in cathode in vol %), as well as (v) the potential vs Li^+^/Li (in V) (Figure 5). For the analysis, a default value was chosen for each parameter, indicated by the colored points. Then, each of the parameters was varied individually, indicated by the colored lines, while keeping the other parameters constant at their default values.

**Figure 5:**
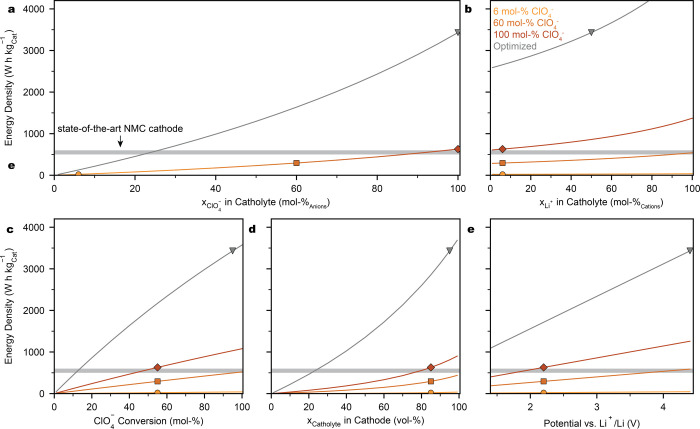
Achievable energy densities of perchlorate cathodes coupled with a Li metal anode. (a–e) Gravimetric energy densities of Fe@C cathodes infiltrated with a Li/Pyr_1,4_/TFSI/ClO_4_ catholyte depending on the ClO4^–^ (a) and Li^+^ (b) fraction in the Li/Pyr_1,4_/TFSI/ClO_4_ catholyte, the degree of ClO4^–^ conversion (c), the fraction of catholyte within the cathode (d), and the cell potential (e). The colored lines indicate the isolated impact of each parameter on the energy density for different cell architectures, with all other values being kept constant at their default values. Colored points mark the cell-architecture-specific default values.

For a cathode with the current architecture (6 mol %_Catholyte_ ClO_4_^–^, 6 mol %_Catholyte_ Li^+^, 55 mol % ClO_4_^–^ conversion and 85 vol % porosity, 2.2 V), the bottleneck for improving the energy density is the ClO_4_^–^ concentration, while changing any of the other parameters does not meaningfully increase the energy density (Figure 5).

Increasing the ClO_4_^–^ concentration could only be achieved in the current architecture by increasing the LiClO_4_ concentration of the catholyte. However, an increased LiClO_4_ concentration would result in a highly viscous catholyte or undissolved LiClO_4_, resulting in poor ClO_4_^–^ accessibility to the electrocatalyst. In order to increase the ClO_4_^–^ concentration while maintaining a low-viscosity catholyte in which ClO_4_^–^ is fully dissolved, the anion ratio (ClO_4_^–^/TFSI^–^) should be increased independently of the cation ratio (Li^+^/Pyr_1,4_^+^), since the viscosity and solubility limit is primarily limited by the Li^+^ concentration relative to the sterically more bulky Pyr_1,4_^+^ cation. Hence, the synthesis of Pyr_1,4_ClO_4_ as a catholyte (co)­solvent could provide a compelling avenue to decouple cation and anion ratios, and enable high ClO_4_^–^ concentrations, while maintaining a low-viscosity fully dissolved catholyte through low Li^+^ concentrations.

By adding Pyr_1,4_ClO_4_ and thereby increasing the ClO_4_^–^ concentration to 60 mol %_Catholyte_ would increase the energy density by ca. 15× from 22 W h kg^–1^ to 294 W h kg^–1^. Using a pure LiClO_4_/Pyr_1,4_ClO_4_ catholyte would even result in an energy density of 572 W h kg^–1^, on par with state-of-the-art Lithium nickel manganese cobalt oxide CAMs.

Moreover, if the current cathode architecture was further optimized (100 mol %_Catholyte_ ClO_4_^–^, 50 mol %_Catholyte_ Li^+^, 95 mol % ClO_4_^–^ conversion and 95 vol % porosity, 4.4 V), the energy density could be increased to 3431 W h kg^–1^, far exceeding any theoretically achievable values with state-of-the-art or conversion-type CAMs. On a cell level, the achievable energy density would be further reduced by ca. 50% due to inactive components,^17^ resulting in ca. 1700 W h kg^–1^, more than three to five times more than any other primary Lithium-based battery technologies (Table S2).^18^

By comparison, gasoline, the most common primary energy carrier for transportation, has a theoretical energy density of 12000–13000 W h kg^–1^. However, the ca. 3× lower tank-to-wheel efficiency compared to electric vehicles^19^ means that an equivalent battery electric vehicle would only have to deliver an effective energy density of ca. 3800 W h kg^–1^.

We note that a significant energy gain is expected if the voltage gap between the theoretical and observed cell voltage (4.4 vs 2.2 V) can be narrowed down. The voltage gap arises since the measured cell voltage arises from the TMO_*x*_/TM and not from the ClO_4_^–^/Cl^–^ redox couple while the remaining Gibbs free energy is dissipated as heat. By choosing a TM catalyst with a higher redox potential, this gap could be narrowed down.

## Conclusions

In this work we introduced electrochemically coupled OAT as a new design paradigm to activate inert oxoanions in next-generation LIB CAMs. By using TM NPs as the electrocatalyst in a SSB, LiClO_4_ can be efficiently reduced, resulting in an unprecedented capacity of 1150 mA h g^–1^_LiClO4_, and an energy density of 1950 W h kg^–1^ against a Li metal anode. By performing a sensitivity analysis, we further showcase that the usage of ClO_4_^–^-containing ILs, as well as TMO_*x*_ catalysts with a higher discharge potential are crucial to enhance the energy density on an electrode level to up to 3431 W h kg^–1^.

## Supplementary Material



## Notes

Materials, synthesis details, experimental methods, and supplementary figures and tables (PDF)
