# Supplementary material for: Unlocking Anion Reduction of Lithium Perchlorate via Electrochemically Coupled Oxygen Atom Transfer
Source: J Am Chem Soc. 2026 Jul 14;148(29):30754–61. doi: 10.1021/jacs.5c18724 (PMC13426302; doi:10.1021/jacs.5c18724)
Supplement: Supplementary file 1 [file ja5c18724_si_001.pdf]

Supplementary Materials for

Unlocking Anion Reduction of Lithium Perchlorate via Electrochemically Coupled Oxygen Atom Transfer

Julian F. Baumgärtner,[1,2] Archita Vijay,[1,2] Matthias Klimpel,[1,2] Dmitry Chernyshov,[3] Wouter van Beek,[3] Maksym V. Kovalenko[1,2*] and Kostiantyn V. Kravchyk,[1,2*]

[1]Laboratory of Inorganic Chemistry, Department of Chemistry and Applied Biosciences, ETH Zürich, CH-8093 Zürich, Switzerland.

[2]Laboratory for Thin Films and Photovoltaics, Empa - Swiss Federal Laboratories for Materials Science & Technology, CH-8600 Dübendorf, Switzerland.

[3]Swiss–Norwegian Beam Lines at the European Synchrotron Radiation Facility, 38000 Grenoble, France.

*Corresponding authors. Emails: Kostiantyn.Kravchyk@empa.ch and mvkovalenko@ethz.ch

Methods

Chemicals

Iron(III) nitrate nonahydrate (≥98%, Sigma-Aldrich), poly(VinylPyrrolidone) (PVP, Thermo Scientific, M_w =1.300.000), nickel(II) nitrate hexahydrate (≥99.9%, Strem Chemicals) cobalt(II) nitrate hexahydrate (≥99%, Fluka), hydrochloric acid (37%, VCW Chemicals BDH), Al-doped lithium lanthanum zirconium oxide (LLZO, 99.9%, 500 nm particle size, Ampcera), Carbon black (CB, Super C65, TIMCAL), polymer separator (Celgard-2400), Poly(vinylidene fluoride) (pVdF, average Mw ~534 g mol^{-1}, Sigma-Aldrich), N-Methyl-2-pyrrolidone (NMP, 99%, Roth), Lithium perchlorate (99%, anhydrous, abcr), Lithium bis(trifluoromethanesulfonyl)imide (LiTFSI, 99+%, Solvionic), 1-Butyl-1-methylpyrrolidinium bis(trifluoromethylsulfonyl)imide ($Pyr_{1,4}TFSI$, 99%, iolitec, dried over molecular sieves prior to use), Li rod (99.9%, 12.7 mm Ø, Sigma-Aldrich)

Synthesis

Synthesis of Metal Nitrate/PVP Composites

The synthesis of the iron nitrate/PVP composite was adapted from a previous report.[1] Iron(III) nitrate nonahydrate (3.7 mmol, 1.5 g) and PVP (0.9 g) were dissolved in deionised water (100 ml) at room temperature (RT) by stirring at 1000-1400 rpm until the precursors were fully dissolved, followed by 2h at 500 rpm, resulting in a yellow solution (Figure S1a). The water was evaporated at 90 °C overnight under stirring at 200 rpm, resulting first in a brown solution and eventually a brown iron nitrate/PVP solid composite (Figure S1a).

The same synthesis was adopted for Ni@C and Co@C samples, using nickel(II) nitrate hexahydrate powder (3.7 mmol, 1.079 g) and cobalt(II) nitrate hexahydrate (3.7 mmol, 1.08 g) (Figure S1b).

Synthesis of Carbon-Supported Transition Metal Nanoparticle Catalysts

The synthesis of TM@C was adapted from a previous report.[1] The as-synthesised metal nitrate/PVP composite (~1.2 – 1.5 g) was loaded into an alumina crucible and covered with a lid. The curicple was placed in a tube furnace (Carbolite Gero) and purged with Ar gas for 30 min. Then, the sample was heated to 700 °C in Ar at a heating rate of 3 °C min^{-1}, and held for 3 h before cooling down at 3 °C min^{-1}. The as-synthesized TM@C product was isolated as a black powder, and immediately transferred to an Ar-filled glove box (GB) to minimise air exposure.

Synthesis of Transition Metal Free Carbon Support

As-synthesized Fe@C (ca. 1.2 g) was stirred in hydrochloric acid (~1 M) at 100 rpm for 6 h to dissolve the Fe NPs. The remaining carbon powder was filtered and washed multiple times using deionised water until the wash water was at pH = 7. Then, the powder was collected and dried under vacuum and stored in an Ar-filled GB.

X-ray Diffraction

Powder X-Ray Diffraction

Powder XRD patterns were collected at RT on a Stoe STADI P powder X-ray diffractometer (Cu Kα1 radiation, λ = 1.540598 Å, focusing germanium monochromator) equipped with a Dectris Mythen 1 K silicon strip detector. Samples were prepared in a 0.5 mm Ø borosilicate glass capillary, sealed under Ar and measured in Debye-Scherrer geometry.

Synchrotron X-Ray Diffraction

SXRD data were obtained at the BM01 beamline at the European Synchrotron Radiation Facility. At BM01, the X-ray beam (λ = 0.71792 Å) was adjusted with a sagitally focusing Si(111) double crystal monochromator and a set of collimating and vertically Rh-coated Si mirrors to a beam size

of *ca*. 0.1 mm x 0.35 mm. Diffraction patterns were collected in transmission mode using a Pilatus 2M area photon-counting detectors, and azimuthally integrated using BUBBLE.[2] Patterns were acquired in a 2θ range of 0.5 – 50 ° with a step size of 0.0025° at RT. Samples were prepared in a 0.5 mm Ø borosilicate glass capillary and sealed under Ar.

Synchrotron X-Ray Total Scattering

X-ray total scattering data was obtained at the BM31 beamline at the European Synchrotron Radiation Facility. The X-ray beam (λ = 0.25995 Å) was monochromatized using a liquid nitrogen double-crystal monochromator equipped with a pair of flat Si(111) crystals. Si mirrors to a final beam size of *ca*. 0.3 mm x 0.3 mm. Diffraction patterns were collected in transmission mode on a Pilatus 2M area pixel-counting detectors, and azimuthally integrated using pyFAI.[3] Patterns were acquired for 20 min in a 2θ range of 0.5 – 70 ° (0.5 – 27 $Å^{-1}$) with a step size of 0.014 ° at RT. Samples were prepared in a 0.5 mm Ø borosilicate glass capillary and sealed under Ar. The total scattering data was converted into real space and normalized using PDFgetX3 (*Q*-range 0.5 – 27 $Å^{-1}$).[4] PDF fitting was performed using PDFgui.[4]

Rietveld Refinement and Stacking Fault Modelling

Rietveld refinement was performed with the GSAS-II program.[5] Instrumental parameters were determined with a LaB_6 NIST standard prepared with the same setup that was used for real measurements. The background was modelled by a Chebyschev inverse polynomial The refined values are summarized in Table S1. The background was again modelled by a Chebyschev polynomial with 10 coefficients. Stacking faults were modelled using the DIFFaX code as implemented in GSAS-II.[6]

Thermogravimetric Analysis

The TM@C sample was weighed inside an Ar-filled GB and loaded into an alumina crucible and transferred into a tube furnace (Carbolite Gero). The sample was heated to 550 °C in oxygen at a heating rate of 20 °C min^{-1}, and held for 1 h before cooling down at 20 °C min^{-1}. After subsequent weighing, the amount of TM and C in the original sample was recalculated, assuming complete combustion of carbon and oxidation of the TM to its respective TMO_x.

Scanning Electron Microscopy

SEM images were recorded on the ZEISS Gemini SEM 460 (2 – 10 kV acceleration voltage, 100 – 500 pA beam current, 2 – 11 mm working distance). Energy-dispersive X-ray spectroscopy was performed with an EDS Ultim Max 170 detector of the Zeiss Gemini 460 (10 kV acceleration voltage, 500 pA beam current).

Electrochemistry

Preparation of LLZO SE Pellets

The preparation of LLZO SE pellets was adapted from previous reports.[7-10] 0.5 g of commercially sourced LLZO powder was pressed into 16 mm diameter pellets using a hydraulic pellet-dye press (PerkinElmer), at 3 MPa pressure for *ca.* 10 s. The obtained pellet was polished using sandpaper (P800 Silicon carbide WS Flex waterproof, Hermes) to remove any metallic impurities. The pellet was then heat treated at 200 °C in a tube furnace (Carbolite Gero), for a minimum of 30 mins to remove before taking it into the Ar-filled GB. The pellets were then heated to 900 °C at *ca.* 100 °C min^{-1} in an oven (Nabertherm), followed by a natural cooling step. This step ensures the removal of LiOH and Li_2CO_3 from the surface of the grains.[9] The pellets were then densified in a custom utra-fast fintering setup inside the GB,[8] where the pellet was placed within a BN plate, C-

foil sandwich as shown in ref.[10], in a two-step heating program of 20 s at 1000°C and 120 s at 1200°C. The sintered pellet was once again subjected to an Ar heat treatment process to equilibrate the Li ions, after which it was ready for the subsequent assembly process.[9]

Preparation of Dry Cathodes

The entire cathode preparation was carried out under Ar atmosphere. In a typical cathode preparation, a slurry was prepared from TM@C (100 mg, 70 wt%), CB (29 mg, 20 wt%) and a solution of 0.833 wt-% pVdF binder in NMP (14 mg, 10 wt-% pVdF; 1714 mg, 1200 wt-% NMP). The slurry was mixed under air in a ZrO_2 beaker (20 mL) with ZrO_2 balls (20 g, 3 mm Ø) and ball-milled in a planetary ball-mill (Fritsch, Pulverisette 7) for 1 h at 200 rpm. The slurry was then immediately tape-casted onto carbon-coated Al foil with a doctor blade of 500 µm to obtain a uniform tape. The tape-casted Al foil was dried under Ar at 125 °C for 18 h. 8 mm Ø disks were punched out of the foil and weighed inside GB.

Preparation of Catholyte Infiltrated Cathodes

Safety note: Perchlorate-containing compounds are potentially hazardous due to their strong oxidizing character and associated risk of energetic decomposition. All experiments involving $LiClO_4$ were therefore conducted under Ar atmosphere on small scales (<10 mg), to minimize the risk of explosion.

The entire catholyte infiltration was carried out under Ar atmosphere. To precisely control the $LiClO_4$ loading on the dry TM@C cathode, a 0.1 M solution of $LiClO_4$ in dimethyl carbonate (DMC) was prepared and drop cast onto the cathode, followed by DMC evaporation under vacuum. The $LiClO_4$ loading was determined from the volume of DMC solution. For the reference cell containing LiTFSI, the same procedure was carried out using LiTFSI instead of $LiClO_4$. Next,

$Pyr_{1,4}TFSI$ was drop cast onto the cathode. The volume of IL was calculated based on the free pore volume within the cathode.

Preparation and Assembly of ASSBs containing Catholyte Infiltrated Cathodes

The electrodes were prepared by rolling small pieces of Li cut into a foil approximately 60 µm thick, followed by cutting the foil into 8 mm Ø circular discs. The Li discs was then pressed on one side of the LLZO pellet, while the other side was covered with Al foil to prevent direct contact between LLZO and the medical protection cover in which the Li|LLZO|Al cell was placed for subsequent preparation. The medical protection cover containing the Li|LLZO|Al cell was evacuated, sealed inside the Ar-filled GB, and isostatically pressed (350 MPa, 2 min) using a PW 100 EH cold isostatic press (P/O/Weber) to establish good contact between Li and LLZO. The protective Al foil was then removed from the cold-pressed pellets inside the GB. Next, a polymer separator disc (10 mm Ø) was punched out and placed on top of the previously prepared catholyte infiltrated cathode to prevent spilling of the catholyte. An additional 2 mg IL was drop cast onto the separator to ensure complete wetting. The LLZO|Li pellet was then placed onto the separator with the bare LLZO side facing the separator.

Electrochemical Measurements

Cells were placed within a stainless steel coin cell casing and electrically contacted using a custom pin holder setup. After heating the cell at 125 °C for 24 h, galvanostatic discharge was performed on a multichannel potentiostat/galvanostat from Biologic (MPG2) to a lower cutoff voltage of 1.6 V at a current density of 40 mA g^{-1}_{LiClO4} (ca. 6 µA cm^{-2}). The same areal current density was used for the reference measurements containing LiTFSI.

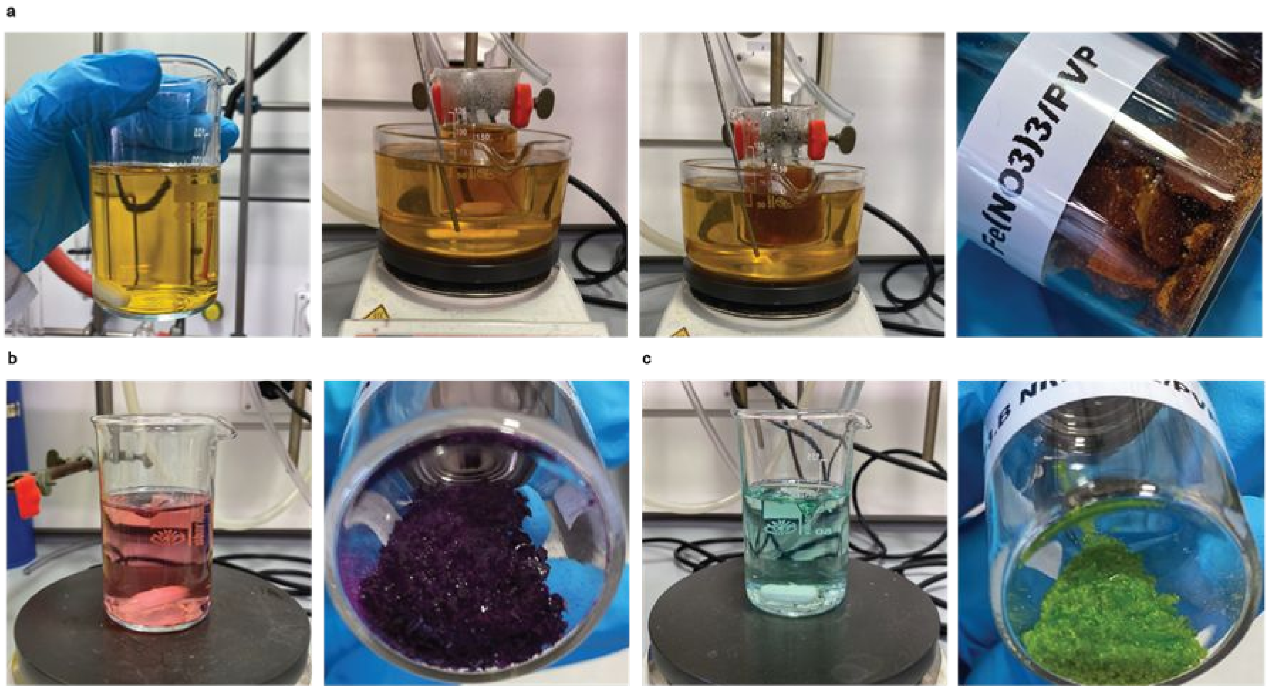

Figure S1. Fotographs of the synthesis of the metal nitrate/PVP composite for Fe (a), Co (b) and Ni (c).

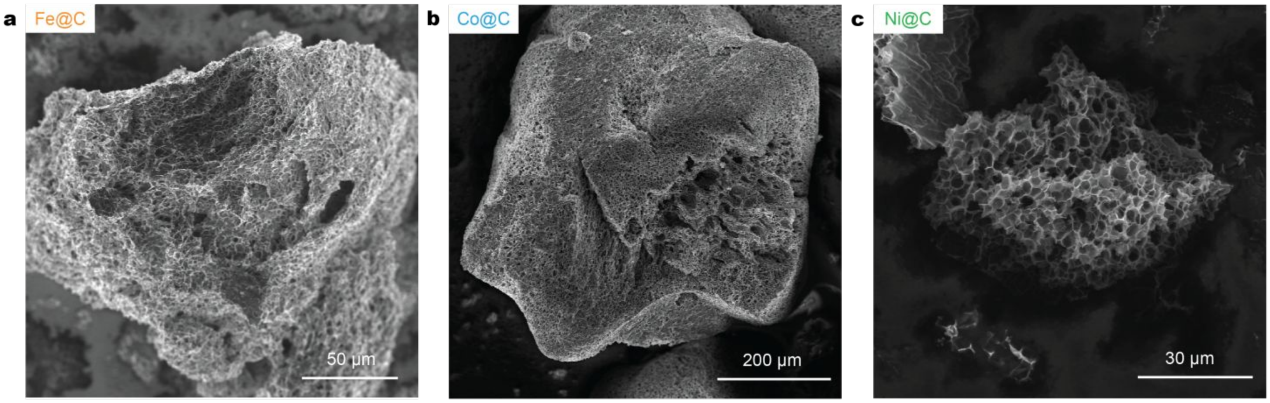

Figure S2. SEM micrographs of Fe@C (a), Co@C (b) and Ni@C (c).

Table S1. Overview of the Rietveld-refined parameters for Fe@C presented in Figure 2b. Values in brackets indicate estimated standard error. If no error is given, the parameter was not refined. U_{iso} was constrained for a given element.

			Fe_3C			
S.G. *Pnma*[11]		x = 93.19(6) wt.-%		d_{cryst} = 97.01(9) nm	ε_{micro} = 0.176(3)%	
a = 5.09191(3) Å		b = 6.74450(4) Å		c = 4.52714(3) Å	$\alpha = \beta = \gamma = 90°$	
Site	Wyck.	x	y	y	Occ.	U_{iso} (Å^2)
Fe1	8d	0.17994(6)	0.06686(5)	0.33459(9)	1	0.0009(1)
Fe2	4c	0.03697(7)	¼	0.83862(16)	1	0.0009(1)
C1	4c	0.8831(7)	¼	0.4485(7)	1	0.0176(9)
N(Reflections) = 135				R_F = 2.25%		
			Fe			
S.G. $Im\overline{3}m$[12]		x = 6.81(6) wt.-%		d_{cryst} = 0.167(10) µm	ε_{micro} = 0.206(10)%	
$a = b = c$ = 2.86752(2) Å				$\alpha = \beta = \gamma = 90°$		
Site	Wyck.	x	y	y	Occ.	U_{iso} (Å^2)
Fe1	2a	0	0	0	1	0.0032(3)
N(Reflections) = 5				R_F = 1.05%		
N(Obs.) = 13903	*N*(Params.) = 31	χ^2 = 12195		GOF = 0.94	R_w = 3.28%	

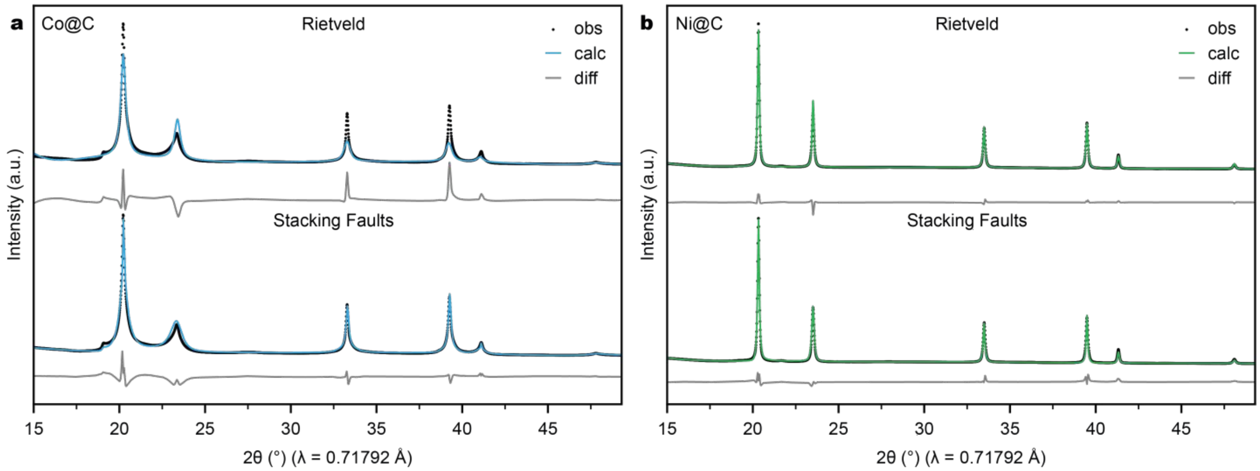

Figure S3. Comparison of Rietveld and stacking faults analysis of SXRD data for Co@C (a) and Ni@C (b).

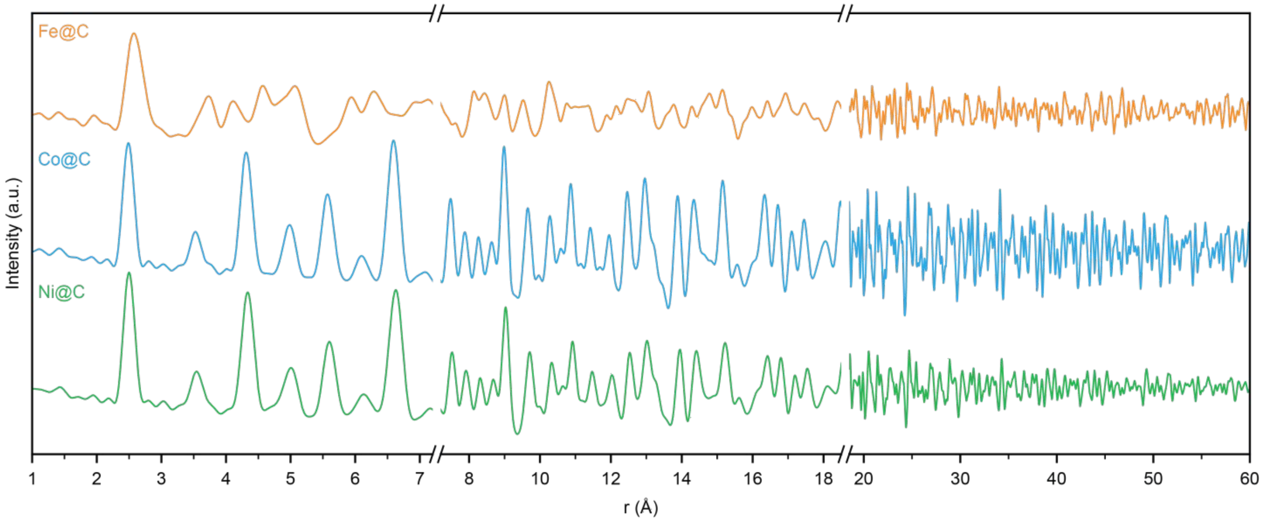

Figure S4. SPDF data for Fe@C, Co@C and Ni@C. The absence of a characteristic metal oxide atom pair correlation (ca. 2.1 – 2.2 Å) excludes any quantitative amorphous metal oxide residues in any of the three samples.

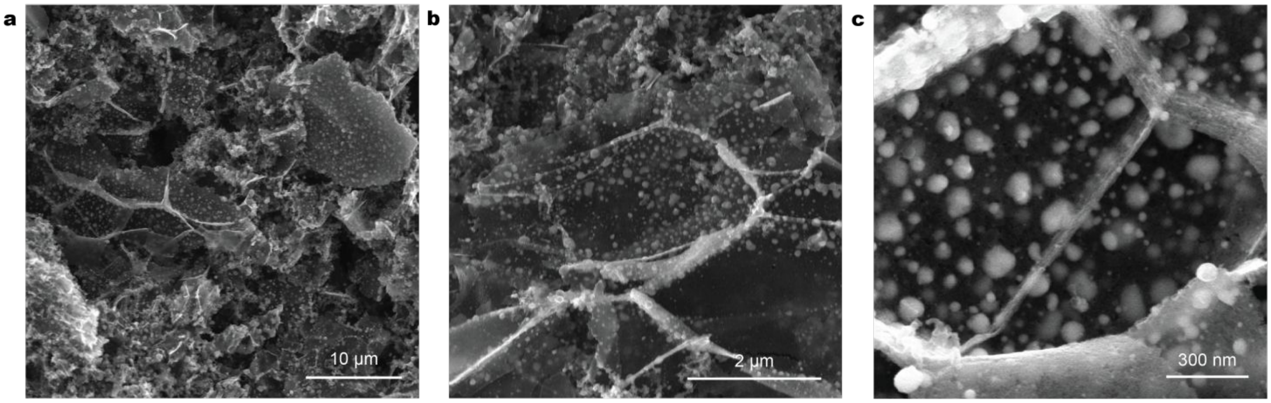

Figure S5. SEM micrographs of Fe@C Cathodes at different magnifications

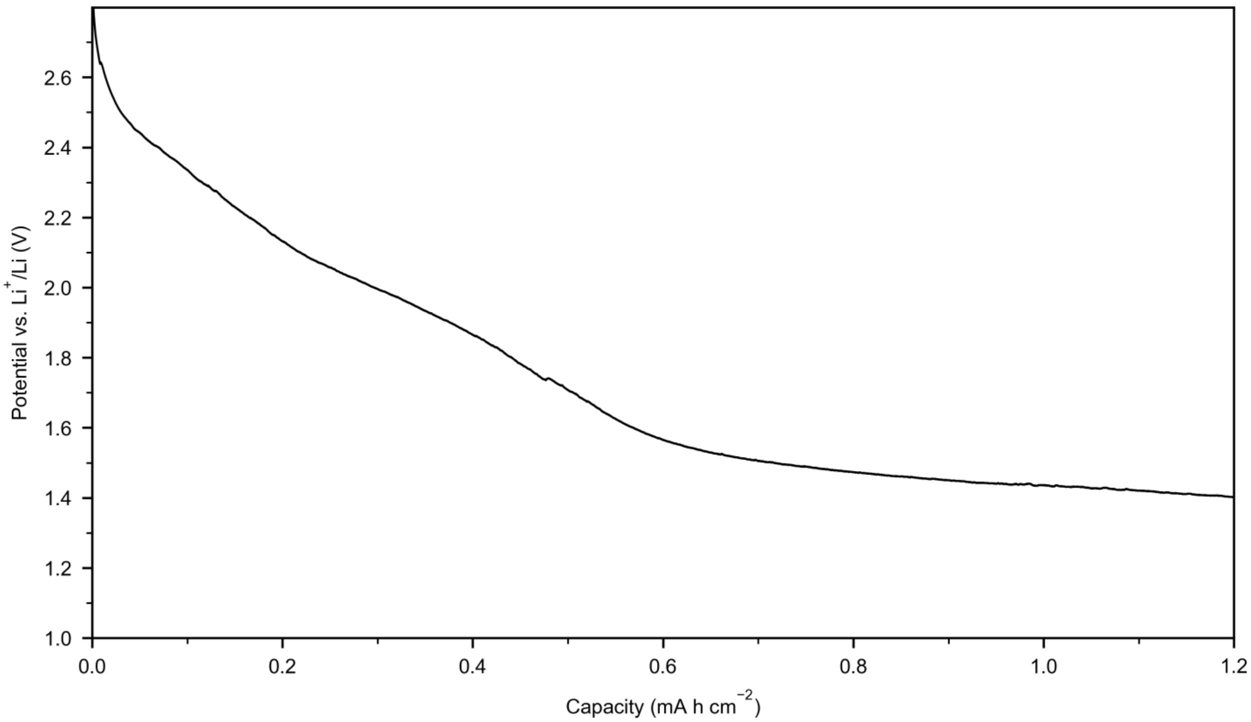

Figure S6. Discharge profile of SSBs with cathodes containing Fe@C catalysts at deep discharge.

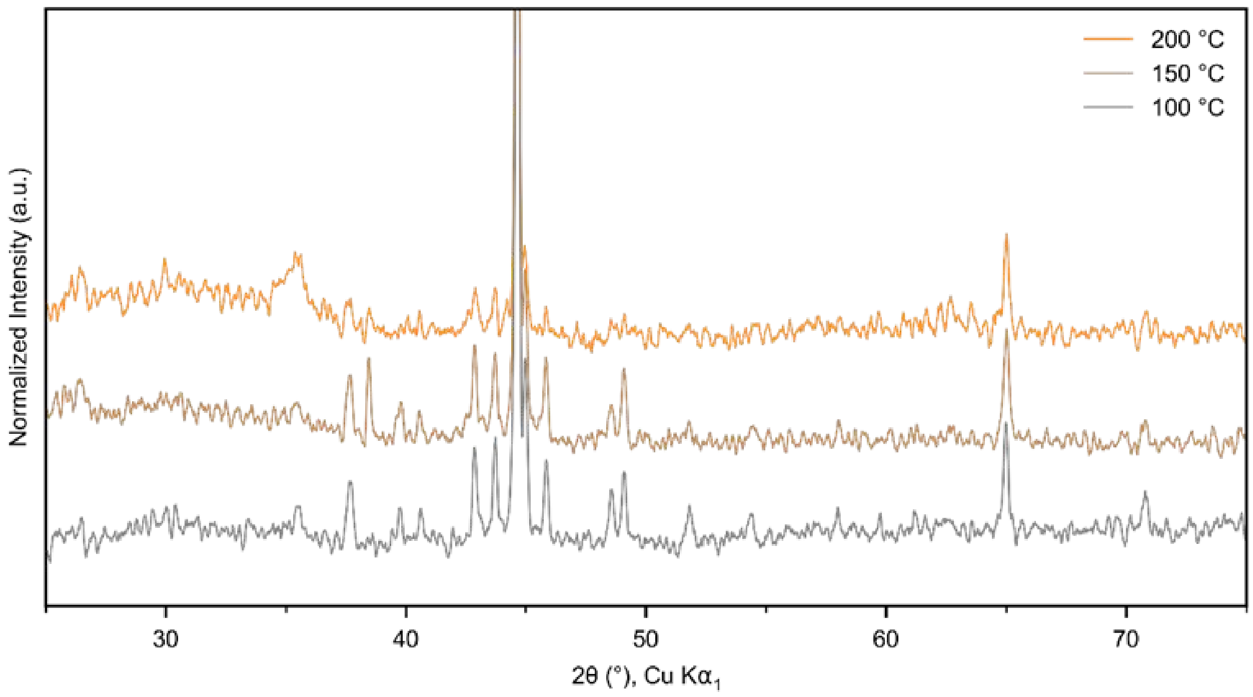

Figure S7. XRD patterns of Fe@C cathodes infiltrated with $LiClO_4$, and heated to different temperatures for 1 h. Fe_3O_4 formation is only observed above 150 °C under these conditions. By extending the heating period in the electrochemically measured cells to 24 h plus the additional discharge time, the operating temperature could be further reduced to 125 °C.

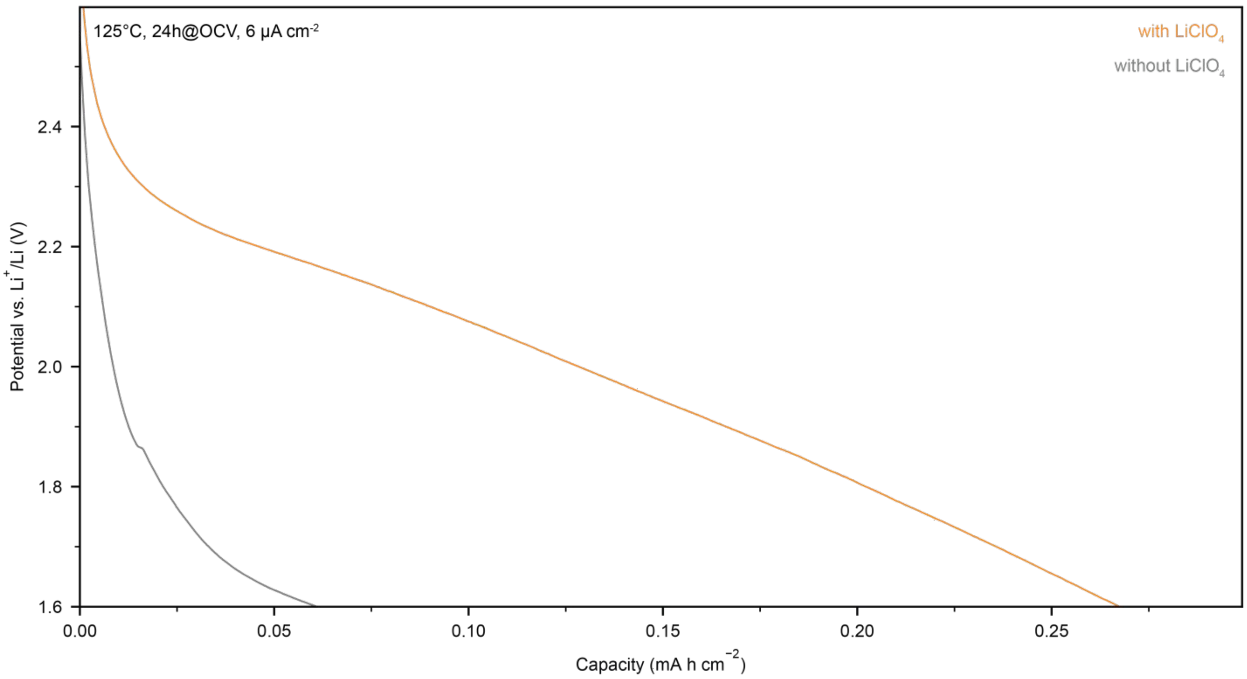

Figure S8. Discharge profile of SSBs with cathodes containing Fe@C catalysts infiltrated with either i) 0.2 mg cm^{-2} $LiClO_4$ in 12 mg cm^{-2} $Pyr_{1,4}$TFSI (orange) or ii) 0.2 mg cm^{-2} LiTFSI in 12 mg cm^{-2} as a reference (grey).

Table S2. Overview of different primary lithium-based battery technologies.[13]

Battery-Technology	Specific cathode capacity (mA h g^{-1})	Potential vs. Li+/Li (V)	Theoretical energy density vs. Li metal (W h kg^{-1})	Practical cell energy density (W h kg^{-1})
Li-I_2	211	2.8	560	240
Li-MnO_2	308	3	856	250
Li-CF_x	865	3	2120	450
Li-$SOCl_2$	451	3.6	1454	590
Li-SO_2	419	3	1134	300
Li-CuO	670	2.2	1256	300
Li-FeS_2	890	1.8	1302	297
Li-$Ag_2V_4O_{11}$	315	3.2	932	270
Li-$LiClO_4$	2018	4.4	5831	1716*

*Calculated considering the energy density of the optimized cathode (3431 W h kg^{-1}), and the energy density losses incurred from inactive components in accordance with the methodology described in Ref.[14] for an all-solid-state battery.

REFERENCES

(1) Wu, F.; Srot, V.; Chen, S.; Lorger, S.; van Aken, P. A.; Maier, J.; Yu, Y. 3D Honeycomb Architecture Enables a High-Rate and Long-Life Iron (III) Fluoride–Lithium Battery. *Adv. Mater.* **2019**, *31* (43), 1905146. DOI: 10.1002/adma.201905146.
(2) Dyadkin, V.; Pattison, P.; Dmitriev, V.; Chernyshov, D. A new multipurpose diffractometer PILATUS@SNBL. *J. Synchrotron Rad.* **2016**, *23* (3), 825-829. DOI: 10.1107/S1600577516002411.
(3) Ashiotis, G.; Deschildre, A.; Nawaz, Z.; Wright, J. P.; Karkoulis, D.; Picca, F. E.; Kieffer, J. The fast azimuthal integration Python library: pyFAI. *J. Appl. Crystallogr.* **2015**, *48* (2), 510-519. DOI: 10.1107/S1600576715004306.
(4) Juhas, P.; Davis, T.; Farrow, C. L.; Billinge, S. J. L. PDFgetX3: a rapid and highly automatable program for processing powder diffraction data into total scattering pair distribution functions. *J. Appl. Crystallogr.* **2013**, *46* (2), 560-566. DOI: 10.1107/S0021889813005190.
(5) Toby, B. H.; Von Dreele, R. B. GSAS-II: the genesis of a modern open-source all purpose crystallography software package. *J. Appl. Crystallogr.* **2013**, *46* (2), 544-549. DOI: 10.1107/S0021889813003531.
(6) Treacy, M. M. J.; Newsam, J. M.; Deem, M. W. A general recursion method for calculating diffracted intensities from crystals containing planar faults. *Proc. R. Soc. Lond., Ser. A* **1991**, *433* (1889), 499-520. DOI: doi:10.1098/rspa.1991.0062.
(7) Okur, F.; Zhang, H.; Baumgärtner, J. F.; Sivavec, J.; Klimpel, M.; Wasser, G. P.; Dubey, R.; Jeurgens, L. P. H.; Chernyshov, D.; van Beek, W.; et al. Ultrafast Sintering of Dense $Li_7La_3Zr_2O_{12}$ Membranes for Li Metal All-Solid-State Batteries. *Adv. Sci.* **2025**, *12* (2), 2412370. DOI: 10.1002/advs.202412370.
(8) Zhang, H.; Dubey, R.; Inniger, M.; Okur, F.; Wullich, R.; Parrilli, A.; Karabay, D. T.; Neels, A.; Kravchyk, K. V.; Kovalenko, M. V. Ultrafast-sintered self-standing LLZO membranes for high energy density lithium-garnet solid-state batteries. *Cell Rep. Phys. Sci.* **2023**, *4* (7). DOI: 10.1016/j.xcrp.2023.101473.
(9) Zhang, H.; Klimpel, M.; Wieczerzak, K.; Dubey, R.; Okur, F.; Michler, J.; Jeurgens, L. P. H.; Chernyshov, D.; van Beek, W.; Kravchyk, K. V.; et al. Unveiling Surface Chemistry of Ultrafast-Sintered LLZO Solid-State Electrolytes for High-Performance Li-Garnet Solid-State Batteries. *Chem. Mater.* **2024**, *36* (22), 11254-11263. DOI: 10.1021/acs.chemmater.4c02351.
(10) Zhang, H.; Okur, F.; Pant, B.; Klimpel, M.; Butenko, S.; Karabay, D. T.; Parrilli, A.; Neels, A.; Cao, Y.; Kravchyk, K. V.; et al. Garnet-Based Solid-State Li Batteries with High-Surface-Area Porous LLZO Membranes. *ACS Appl. Mater. Interfaces* **2024**, *16* (10), 12353-12362. DOI: 10.1021/acsami.3c14422.
(11) Löhberg, K. Zementit als Substitutionsmischkristall des Austenits. *Archiv für das Eisenhüttenwesen* **1961**, *32* (6), 409-412. DOI: 10.1002/srin.196103235.
(12) Hull, A. W. A New Method of X-Ray Crystal Analysis. *Phys. Rev.* **1917**, *10* (6), 661-696. DOI: 10.1103/PhysRev.10.661.
(13) Placke, T.; Kloepsch, R.; Dühnen, S.; Winter, M. Lithium ion, lithium metal, and alternative rechargeable battery technologies: the odyssey for high energy density. *Journal of Solid State Electrochemistry* **2017**, *21* (7), 1939-1964. DOI: 10.1007/s10008-017-3610-7.

(14) Betz, J.; Bieker, G.; Meister, P.; Placke, T.; Winter, M.; Schmuch, R. Theoretical versus Practical Energy: A Plea for More Transparency in the Energy Calculation of Different Rechargeable Battery Systems. *Advanced Energy Materials* **2019**, *9* (6), 1803170. DOI: 10.1002/aenm.201803170.
